# Smartphone Apps for Diabetes Medication Adherence: Systematic Review

**DOI:** 10.2196/33264

**Published:** 2022-06-21

**Authors:** Sheikh Mohammed Shariful Islam, Vinaytosh Mishra, Muhammad Umer Siddiqui, Jeban Chandir Moses, Sasan Adibi, Lemai Nguyen, Nilmini Wickramasinghe

**Affiliations:** 1 Institute for Physical Activity and Nutrition School of Exercise and Nutrition Sciences, Faculty of Health Deakin University Melbourne Australia; 2 College of Healthcare Management and Economics Gulf Medical University Ajman United Arab Emirates; 3 Department of Internal Medicine Sidney Kimmel Medical College Thomas Jefferson University Philadelphia, PA United States; 4 School of Information Technology Deakin University Burwood Australia; 5 Iverson Health Innovation Research Institute Swinburne University of Technology Melbourne Australia

**Keywords:** smartphones, digital health, diabetes, medication adherence, applications, apps, mHealth, mobile health, task-technology fit

## Abstract

**Background:**

Diabetes is one of the leading noncommunicable chronic diseases globally. In people with diabetes, blood glucose levels need to be monitored regularly and managed adequately through healthy lifestyles and medications. However, various factors contribute to poor medication adherence. Smartphone apps can improve medication adherence in people with diabetes, but it is not clear which app features are most beneficial.

**Objective:**

This study aims to systematically review and evaluate high-quality apps for diabetes medication adherence, which are freely available to the public in Android and Apple app stores and present the technical features of the apps.

**Methods:**

We systematically searched Apple App Store and Google Play for apps that assist in diabetes medication adherence, using predefined selection criteria. We assessed apps using the Mobile App Rating Scale (MARS) and calculated the mean app-specific score (MASS) by taking the average of app-specific scores on 6 dimensions, namely, awareness, knowledge, attitudes, intention to change, help-seeking, and behavior change rated on a 5-point scale (1=strongly disagree and 5=strongly agree). We used the mean of the app’s performance on these 6 dimensions to calculate the MASS. Apps that achieved a total MASS mean quality score greater than 4 out of 5 were considered to be of high quality in our study. We formulated a task-technology fit matrix to evaluate the apps for diabetes medication adherence.

**Results:**

We identified 8 high-quality apps (MASS score≥4) and presented the findings under 3 main categories: characteristics of the included apps, app features, and diabetes medication adherence. Our framework to evaluate smartphone apps in promoting diabetes medication adherence considered physiological factors influencing diabetes and app features. On evaluation, we observed that 25% of the apps promoted high adherence and another 25% of the apps promoted moderate adherence. Finally, we found that 50% of the apps provided low adherence to diabetes medication.

**Conclusions:**

Our findings show that almost half of the high-quality apps publicly available for free did not achieve high to moderate medication adherence. Our framework could have positive implications for the future design and development of apps for patients with diabetes. Additionally, apps need to be evaluated using a standardized framework, and only those promoting higher medication adherence should be prescribed for better health outcomes.

## Introduction

Diabetes is one of the leading noncommunicable chronic diseases globally and poses a significant challenge to individuals’ physical and mental health and quality of life [1,2]. According to the International Diabetes Federation, in 2019, approximately 463 million adults (9.3% of the global adult population) aged 20-79 years lived with diabetes [3,4]. By 2045, the number of people with diabetes is projected to surge to 700 million [3,4]. People with diabetes face long-term disease burden and financial costs [5], and an estimated 79% of adults with diabetes live in transitional countries [3]. In addition, more than 1.1 million children and adolescents are living with type 1 diabetes mellitus (T1DM) [3], involving autoimmune destruction of pancreatic β-cells, resulting in insulin deficiency [6]. Aging, urbanization, and lifestyle factors are causative factors of type 2 diabetes mellitus (T2DM) [4], which is a chronic metabolic disorder characterized by insulin insensitivity as a result of insulin resistance, declining insulin production, and eventual pancreatic β-cell failure [7]. Further, deaths due to diabetes have reached an alarming 4.2 million annually [3].

In patients with diabetes, blood glucose (BG) levels need to be monitored regularly and managed adequately to maintain health and well-being [8]. Nevertheless, almost half of the people with diabetes remain nonadherent to their prescribed medications [9-11], leading to uncontrolled diabetes, poor outcomes, and lower quality of life [10,12-14]. Nonadherence to medical therapy also results in increased absenteeism, hospitalization risk, and need for health care, which have an enormous economic impact on individuals and society [15,16]. Several factors contribute to poor adherence to medication, including complex dosing regimens, clinical inertia, safety concerns, socioeconomic issues, costs of medication, ethnicity, patient education and beliefs, social support, and polypharmacy [13,17-19]. The Prospective Urban Rural Epidemiology (PURE) study showed that 26.9% and 63.0% of households in low-income countries could not afford metformin and insulin, respectively [20]. High adherence to diabetes treatment has a beneficial impact on BMI, lipid and glycemic control, and emotional and physical performance [21]. Younger age, higher numbers of medications, and higher hemoglobin A_1c_ levels have been associated with lower medication adherence among patients with T2DM [22].

The widespread applications of information and communication technologies (ICTs) in the health sector have resulted in significant improvements in the health care delivery system, such as promoting patient-centered health care, improving quality of care, and educating health professionals and patients [23]. ICTs, including web-based, mobile phone–based, and digital technologies for electronic capture, storage, processing, and information exchange, have been used to prevent and manage chronic disease and improve medication adherence [5,24-27]. Several studies have shown mobile health as an effective and cost-effective approach for improving diabetes care [5,28-30]. The Global Observatory for eHealth, World Health Organization, defines mobile health (mHealth) as “medical and public health practice supported by mobile devices, such as mobile phones, patient monitoring devices, personal digital assistants, and other wireless devices.” mHealth capitalizes on mobile phones’ core utility of voice and SMS as well as more complex functionalities and applications, including general packet radio service, mobile telecommunications, a global positioning system, and Bluetooth technology [31].

Smartphone apps on diabetes management and self-management are available for the public to download from major app stores, including Google Play and Apple App Store [32,33]. Further, several apps are available, which can collect health data, providing clinical decision support systems and assisting with medication adherence [34]. mHealth interventions are promising for the treatment and management of diabetes [35]. Further, there is strong evidence for the efficacy of apps for lifestyle modification in patients with T2DM [36] and self-managed tasks in improving health outcomes [37]. Apps have been effective in increasing treatment adherence among patients with various conditions, such as asthma, heart failure, hypertension, and HIV [38]; medication adherence among patients with depression, cardiovascular disease, Parkinson disease, hypertension, and multimorbidity [39]; older adults with coronary heart disease [40]; and essential hypertension [41]. Although a few recent reviews evaluated medication adherence among individuals with diabetes [42-44], to our knowledge, no systematic reviews have been undertaken to identify high-quality apps and evaluate their features for diabetes medication adherence. Hence, this study systematically reviews and evaluates apps available for diabetes medication adherence and presents the technical features of high-quality apps freely available to the public in Google Play and Apple App Store.

## Methods

### Design

This study adopted the principles of a systematic review process to identify and select apps, including using an app quality assessment tool, a search strategy, predefined inclusion criteria to screen apps, app rating and selection, and data extraction for qualitative analysis.

### App Selection and Assessment

#### Search Strategy

**Figure 1:**
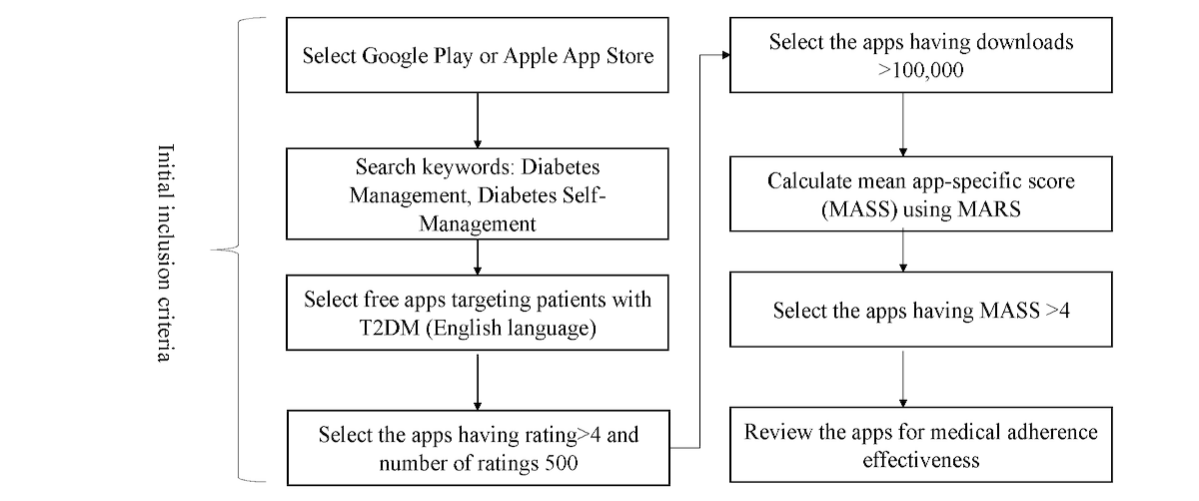
App selection steps followed for Google Play and Apple App Store. MARS: Mobile App Rating Scale, T2DM: type 2 diabetes mellitus.

Globally, the dominating operating systems in the smartphone market are Android and iOS [45]. Hence, we searched Google Play (Android) and Apple App Store (iOS) in May 2020 for apps used for diabetes medication adherence without country-specific restrictions. The key search terms used “Diabetes OR Diabetic OR Diabetics OR blood glucose OR blood sugar” AND “medication OR medicine OR drugs OR medication adherence OR medication support.” The search produced a list of apps for screening. Figure 1 illustrates the process of app selection from the respective app stores.

#### App Screening

Two researchers (VM and MS) screened the app titles and descriptions from the app stores, using the inclusion and exclusion criteria as shown in Textbox 1. In cases of disagreement, the third reviewer (SMSI) intervened to evaluate the situation and reach a consensus. We considered apps available on both the Apple and Google platforms as single apps. Further, we included apps with more than 100,000 downloads to identify the most common apps used in diabetes medication adherence, following the approach of a similar study to assess the quality of apps and perform a content analysis of apps targeting medical adherence [46]. We evaluated high-quality apps using the Mobile App Rating Scale (MARS) [47]. Apps achieving a total mean quality score greater than 4 out of 5 in our study were considered to be of high quality [48]. MARS is a validated tool that measures app quality across 5 multidimensional layers to assess the quality of apps: (1) engagement, (2) functionality, (3) aesthetics, (4) information, and (5) app subjective quality [47]. The MARS scale gives equal weightage to all 5 dimensions and uses a 5-point rating scale from 1 to 5 with 1=inadequate and 5=excellent. The maximum score for each dimension is 25 for engagement, 20 for functionality, 15 for aesthetics, 35 for information, and 20 for app subjective quality. We calculated the total mean score by determining the mean of the average score for the 5 dimensions [47].

**App selection criteria.**

**Inclusion criteria:**

- Apps for adults with type 2 diabetes
- Apps with functionality supporting medication adherence or self-management features
- Apps in the English language
- Apps available for free and not requiring a paid subscription
- Apps with >500 user ratings

**Exclusion criteria:**

- Apps intended only for use by health care professionals and not general public
- Apps not updated since January 1, 2018
- Apps providing only diabetes education or suggestions for medication reminders
- Apps with country restrictions
- Apps that had any technical issues such as problems with downloading, logging in, and crashing

#### App Assessment

To determine which apps to include in this study, the apps obtained after preliminary screening and application of the initial inclusion criteria were downloaded and used by the two authors (VM and MS) independently to test their functionality. After discussion, the quality assessment was reported and in case of disagreements, input from the third author was sought. Further, we calculated the mean app-specific score (MASS) by taking the average of app-specific scores on 6 dimensions, namely, awareness, knowledge, attitudes, intention to change, help-seeking, and behavior change. These are also rated on a 5-point scale with 1=strongly disagree and 5=strongly agree. We used the mean of the app’s performance on these 6 dimensions to calculate the MASS [47]. We further assessed the perceived impact of the app on the users’ knowledge, attitudes, intentions to change, and the likelihood of actual change on the target health behavior (diabetes medication adherence in our case). Multimedia Appendix 1 presents an app-specific evaluation and the MASS for the considered apps.

#### App Rating and Selection

We considered apps with a MASS greater than 4 to identify high-quality apps for diabetes medication adherence.

### Data Extraction

Two reviewers performed the data extraction. One reviewer evaluated Apple App Store’s apps, and the other reviewer evaluated Google Play apps. Before reviewing the apps, the reviewers conferred and decided to include all the critical features that the apps provide. We specifically focused on medication reminders and the adherence features that the apps offer. The reviewers extracted information on the app features using a predetermined Excel (Microsoft Inc) sheet. The Excel sheet was developed on the basis of a pilot app assessment exercise with 5 apps with extensive features; the Excel sheet was refined for this study. We excluded apps that could not be assessed owing to country-specific or other restrictions. We included additional features of free apps that were available upon subscription only.

### Data Analysis

We grouped the apps on the basis of the operating system (ie, Android and iOS), presented the mean MARS rating, and summarized the main features. We also presented the MASS and app-specific rating for medication adherence based on awareness, knowledge, attitude, intention to change, help-seeking, and behavior change.

## Results

### Overview

**Figure 2:**
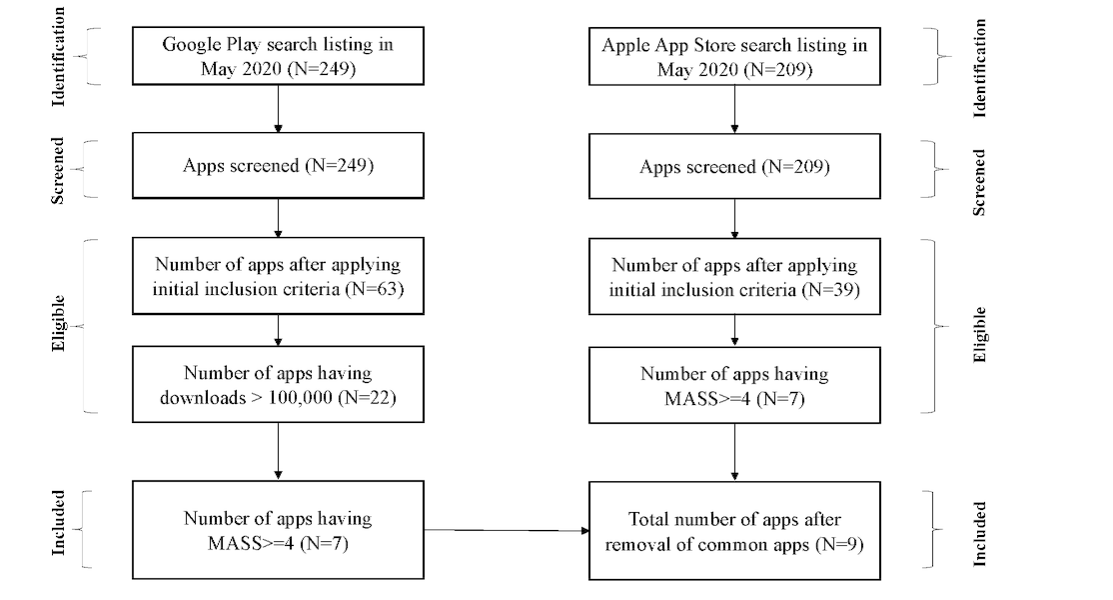
Summary of app selection for review. MASS: mean app-specific score.

We identified 249 apps in Google Play and 209 apps in the Apple App Store. The initial inclusion criteria resulted in 63 and 39 Apps for Google Play and Apple App Store, respectively. Finally, 8 apps with a MARS greater than 4 were included. The mean MASS for the included apps was 4.2, and the average app rating by users was 4.7. Figure 2 depicts the app selection methodology followed for both Apple App Store and Google Play for selecting the relevant apps that satisfy the selection criteria.

### Characteristics of the Included Apps

All 8 apps were available in the Apple Store [49-56], 7 were available in Google Play [49-55], and one app had the option to be accessed through a web application and Amazon Alexa [56]. Given that all the apps were available in the Apple App Store, we extracted information for the apps from the App Store. Table 1 presents an overview of the apps. In contrast, Multimedia Appendix 2 presents detailed information for each app.

**Table 1:** Overview of the apps.

Apps	Operating system	Category	Available languages, n	Data privacy				Seller		In-app purchases
				Identified	Deidentified					
Diabetes:M [49]	Android and iOS	Medical	8	Yes	Yes		Chronic disease software development company		Yes	
mySugr - Diabetes Tracker Log [52]	Android and iOS	Medical	24	Yes	No		Digital health company		Yes	
Health2Sync [51]	Android and iOS	Medical	4	Yes	No		Digital health company		Yes	
MyTherapy Pill Reminder [53]	Android and iOS	Medical	30	No	Yes		Health, wellness, and fitness		No	
One Drop: Transform Your Life [54]	Android and iOS	Health and fitness	11	Yes	Yes		Software and Technology services		Yes	
Glucose Buddy Diabetes Tracker [50]	Android and iOS	Medical	31	Yes	Yes		Individual		Yes	
OneTouch Reveal [55]	Android and iOS	Medical	14	Yes	Yes		Diagnostic systems manufacturer		No	
Sugarmate [56]	iOS	Medical	5	Required to provide with the next app update		Required to provide with the next app update		Software company		No

The Apple App Store apps were categorized as medical [49-53,55,56] and health and fitness [54] apps. Including English language, the apps were available in around 5 [51,56], 10 [49,54,55], 20 [52], and 30 languages [50,53]. Additionally, all the apps had family sharing options (ie, the app could be shared with and used by 6 family members) [49-56]. Apart from one app [56], the apps defined their privacy policy explicitly, defining the identified [49-52,54,55] and deidentified data [49,50,53-55], although it differed between the apps. For example, data such as health and fitness information, contact information, identifiers, diagnostics, location, user content, and usage data were considered “identified” data by one app [52]. In contrast, the same data were considered “deidentified” data by another app [53]. Further, the apps were sold by software companies [54,56], digital health companies [49,51,52,55], health and fitness companies [53], and individuals [50]. Finally, although all the apps were free [49-56], they had an in-app purchase option [49-52,54]; that is, users had the option to pay a prescribed amount to access additional features, such as premium subscription features [49-52] and access to a coach [54].

The compatible operating system (OS) version for the apps varied drastically between the apps. For the apps to run smoothly, 9.0 [49], 10.3 [56], 11.0 [51], 12.0 [50], 13.0 [53-55], 13.2 [52] or later versions of the OS were required.

### App Features

#### Overview

**Figure 3:**
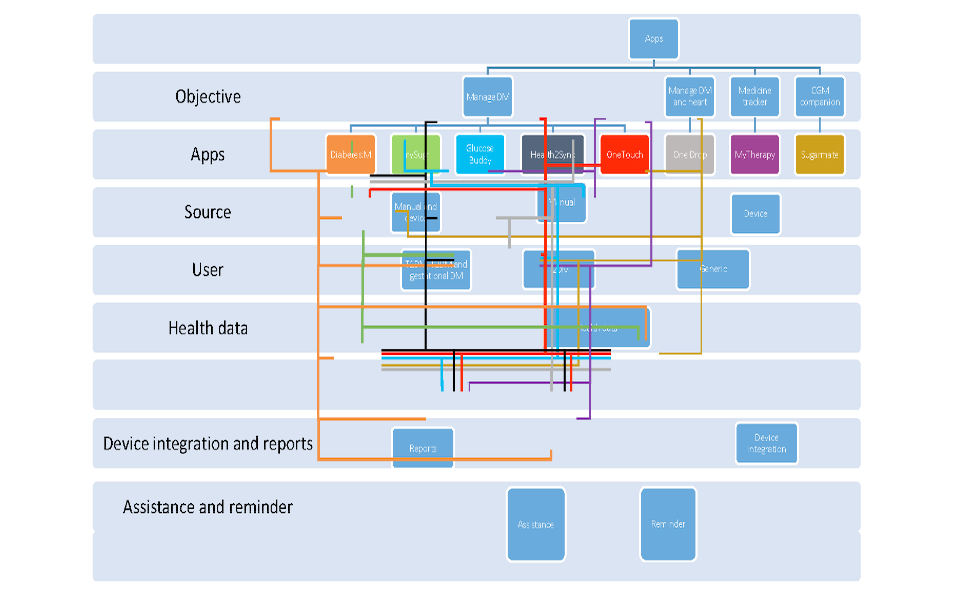
Apps and their corresponding features. CGM: continuous glucose monitoring, DM: diabetes mellitus, T1DM: type 1 diabetes mellitus, T2DM: type 2 diabetes mellitus.

In this section, we discuss the salient features of the apps evaluated against standard parameters. However, we have considered the free app features for this analysis (and we have not considered the in-app features). Figure 3 illustrates the apps and their corresponding features.

#### Device Objective and Target Population

The primary objective of the apps was to manage diabetes [49-52,55], manage diabetes and heart health [54], assist in medication tracking [53], and function as a companion to a continuous glucose monitoring (CGM) device [56]. Although a few apps have been developed for the general population irrespective of their medical condition [50,53,56], a few other apps aimed to address the needs of patients with T2DM [51,54]. In contrast, other apps had functionalities to address the health and well-being needs of patients with T1DM, T2DM, or gestational diabetes mellitus [49,52,55]. The apps had age ratings of 4+ [50-54,56] and 17+ [49,55].

#### BG Reading

The apps had different methodologies and used varying technologies to capture the BG recordings. For example, some apps could log in to the recordings manually [50,53]. In contrast, a few other apps could capture CGM recordings from the integrated BG monitoring (BGM) devices [55,56]. Nevertheless, some other apps could manually log in the BG recordings and CGM recordings from the integrated BGM devices [49,51,52,54].

#### Health Data

In addition to recording BG readings, the apps had the option to capture other health-related data, including food consumed [49,50,52,54-56], blood pressure [50-54], weight [50-54], daily activity and steps walked [52,54-56], and medication [49,50,52,54]. Further, regarding food consumption, the apps specifically considered the intake of carbohydrates [49,50,52,55,56], protein [49], fats [49], and calories [49].

#### Device Integration

There are various BGM or CGM devices available in the market, and some apps could integrate and function with multiple devices, such as Dexcom, OneTouch, and Accu-Chek [49,51,54]. In contrast, other apps functioned exclusively with a device, such as Accu-Chek [52] and Dexcom [49,50,54]. Moreover, an app developed by the seller of the OneTouch device aimed to capture CGM recordings to perform analysis tasks integrating other health parameters [55]. In contrast, a seller not related to the Dexcom device developed an app to capture the CGM readings in real time and perform other tasks [56].

#### Reports

The apps had features to generate reports on BG levels, taking into consideration other factors including food consumed [49,50,52,54-56], physical activity [49,54,55], medication [49,50,52,54], and other vital recordings [50,51,53,54,56]. From the reports generated, the apps provided an overview of short durations of captured data such as whenever the app is launched [51]; hourly [50], daily [49,52,54,56], weekly [49,52,54], and fortnightly [55]; and longer durations such as monthly [49,52,54] and yearly [49]. Moreover, a few apps could alert individuals when their BG levels dropped below or increased past threshold values through notifications [49,55], SMS text messaging [51,56], or phone calls [56].

#### Reminders

Forgetting to take medication is a significant cause of medication nonadherence among patients [57]; hence, apps with reminders could improve adherence. The apps had reminders to check glucose levels [49], take meals [49,54,55], medications [53,54], exercise [53,54], and address other personalized medical needs [51,55].

#### Adherence Motivation

To continuously support individuals in their journey of diabetes medication adherence, apps have provided options to invite clinicians, nutritionists, family, friends, and loved ones to view their progress and accordingly assist them [51,53,54]; have patient information for further support [49], providing personalized tips [51,53,55]; and deliver an education plan featuring 5-minute lessons [50]. In addition, a few apps had gamification features (ie, elements of game-playing), such as point-scoring, competition with others, and challenges to encourage engagement [52,54-56].

### Diabetes Medication Adherence

Among people with diabetes, optimum glycemic control is essential, and good adherence is associated with a lower risk of all-cause mortality and hospitalization [58]. Many models have been studied to explore the acceptance and use of technologies. One such model is task-technology fit (TTF). TTF states that a technology should be effective in completing the assigned task, which will lead to an increase in user performance and adoption [59]. TTF rates the task characteristics and technology characteristics of apps, which affects the effectiveness and adoption of the apps by the user. Accordingly, in this section, we used the TTF and quantified the effectiveness of apps in diabetes adherence to evaluate whether the apps promote adherence to diabetes medication [60,61]. A recent study used TTF to evaluate the effectiveness of the mHealth app in delivering health care services [62].

Primary prevention and mitigating the severity of diabetes revolve around regular BG checks, food consumed, physical activity, other vital readings, and adherence to medication [63]. Hence, they are the primary factors considered in this study. Although apps have been found to improve awareness of medication adherence and reduce self-reported barriers to medication adherence among medication-nonadherent patients with diabetes [64], a study observed that a large proportion of diabetes self-management apps lacked features for enhancing medication adherence and safety [65]. Consequently, among the app features, we considered the overview (report generated and graphically presented for a period), reminders (customized alerts to perform tasks), notifications (alerts when BG shoots up or drops below the threshold), assistance (additional support provided by various stakeholders), and motivation (assistance from loved ones and challenges through gamification) as the imperative app features needed to support an individual in diabetes medication adherence.

Table 2 presents a TTF matrix for diabetes medication adherence to evaluate the apps against the defined primary factors and app features. In the matrix, we have marked “yes” when factors and features are available. We then totaled the score for primary factors and app features separately. We defined a score of 5 in both primary factors and app features as apps assisting in very high adherence, 4 and above in either primary factors or app features as apps assisting in high adherence, and a score of 3 and above in either primary factors or app features as apps assisting in moderate adherence. We further defined a low adherence app as apps scoring 2 and above in either primary factors or app features and any score below 2 for primary factors or app features as apps resulting in poor adherence.

**Table 2:** The task-technology fit matrix for diabetes medication adherence.

App	Primary factors							App features					
	Blood glucose	Food	Physical Activity	Medication	Vital signs	Score	Overview		Reminder	Notification	Assistance	Motivation	Score
Diabetes:M [49]	Yes	Yes	Yes	Yes	No	4	Yes		Yes	Yes	Yes	No	4
mySugr - Diabetes Tracker Log [52]	Yes	Yes	No	Yes	No	3	Yes		No	No	No	Yes	2
Health2Sync [51]	Yes	No	No	No	Yes	2	Yes		Yes	Yes	Yes	Yes	5
MyTherapy Pill Reminder [53]	Yes	No	No	No	Yes	2			Yes	No	Yes	Yes	3
One Drop: Transform Your Life [54]	Yes	Yes	Yes	Yes	Yes	5	Yes		Yes	No	No	Yes	3
Glucose Buddy Diabetes Tracker [50]	Yes	No	No	Yes	Yes	3	Yes		No	No	Yes	No	2
OneTouch Reveal [55]	Yes	Yes	Yes	Yes	No	4	Yes		Yes	Yes	Yes	Yes	5
Sugarmate [56]	Yes	Yes	No	Yes	Yes	4	Yes		No	Yes	No	Yes	3

According to our evaluation, none of the apps assists in very high adherence, and there are no poor-adherence apps. In addition, we found that 3 apps each assisted in high adherence (25%) [49,55] and moderate adherence to diabetes medication (25%) [54,56]. In contrast, 4 other apps were in the low adherence category in assisting with diabetes medication adherence (50%) [50-53].

## Discussion

### Principal Findings

In this systematic review, we identified 8 high-quality apps publicly available in the Apple App Store and Google Play free of charge. The mean MASS of the apps was 4.2, while the average user ratings was 4.7. Only 2 apps showed high adherence and 2 apps showed moderate adherence, while half of the apps showed low adherence. However, since the global market is poised for rapid growth and could have widespread implications in delivering personalized health care [66], interoperable mHealth solutions available in the Apple App Store and Google Play are generally designed and developed for widespread adaptability. Our systematic review suggests evaluating apps using a standardized framework before prescribing them to patients with diabetes, and using behavioral theories for improving medication adherence.

All the apps were available in English and more than 5 other languages [49-56]. Moreover, bilingual English-speaking patients expressed the need for language translation to understand and communicate using the apps [67], and the selected apps could address this concern through the multilanguage option provided. However, globally, 79% of adults with diabetes live in transitional countries [3]. An increase in mobile phone subscriptions over the years, including in transitional countries [68], and the constant technological advancements in mobile phones [69] promote mHealth as effective for use in transitional countries having barriers, such as lack of infrastructure and equipment and technology gaps [70]. Hence, if the selected apps have languages that predominate in transitional countries with a higher prevalence of diabetes, they might assist in diabetes management, reducing the burden on the health care system and curbing mortality rates.

Deidentification is essential for protecting patient privacy and removing identifiers that directly or indirectly point to a person [71]. However, there is variability in the definition of deidentification [72]. For instance, among the considered apps, health and fitness, contact information, identifiers, diagnostics, location, user content, and usage data were considered identified data by one app [52]. In contrast, the same data were deemed deidentified data by another app [53]. This discrepancy could be due to differences in definitions and legal practice followed in countries where the apps have been developed [72]. Hence, health care apps developed in accordance with the guidelines of legislation, such as the US Health Insurance Portability and Accountability Act and the European General Data Protection Regulation, could have uniformity in defining identified and deidentified data [72]. Accordingly, following global regulatory guidelines could bring uniformity in the use of identified and deidentified data.

In-app purchases permit the user to purchase added services from within an app, and 5 apps considered in this study had this feature [49-52,54]. Remarkably, in-app purchase options work well in promoting health apps wherein the essential functions are offered freely [73]. However, the intention to upgrade to a paid subscription is driven by the subscription features, benefits, and price value [74]. Hence, developing free health apps with in-app purchase options will be necessary to promote the app; however, the content should provide sufficient value to retain subscriptions [74].

A few apps described in this study could be used by all patients with diabetes [50,53,56]. In contrast, other apps were specifically targeted to patients with T2DM [51,54] or to those with T1DM, T2DM, or gestational DM [49,52,55]. Nevertheless, apps with specific descriptions were sought more by users compared to those with general descriptions [75]. Some apps had an age rating of ≥4 [50-54,56] and ≥17 [49,55]. T1DM affects children [3], and although some apps were indicated as suitable for patients with T1DM, the age rating was ≥17 [49,55]. Another app had an age rating of ≥4 and could be used by patients with T1DM [52]. Hence, developing apps with specific descriptions and appropriate age ratings could assist the appropriate target population.

We have considered physiological factors influencing diabetes and app features to develop and assess apps using the TTF matrix.

### Limitations of the Research

This review had certain limitations. First, in this study, we included apps that had greater than 500 ratings. Therefore, we could have eliminated several new apps with fewer than 500 reviews, but that might have had a higher MASS. Second, we included only apps in the English language, possibly missing effective apps in languages other than English. Third, our search date was limited to May 2020. However, the constraints associated with the severity of the COVID-19 pandemic could have enabled the development and usage of apps that satisfied our selection criteria. Finally, we limited our search to the apps available in the major app stores: Apple App Store and Google Play. However, these stores account for 80% (4.41 million) of the global apps as of May 2020 [76].

### Implications and Future Research

Our findings provide guidelines for app developers to develop an app that assists in diabetes medication adherence. Further, several apps are very likely to be developed and prescribed during the pandemic period [77,78]. Therefore, evaluating the apps using our framework could help the apps to provide high medication adherence for better health and well-being of patients with diabetes. With the advancement in smartphone technology, various health vitals could be captured and transferred in real time for analysis [79-81]. The application of machine learning and big data could provide a wealth of predictive information to the patient and to health care professionals [82-87]. Furthermore, high-quality apps coupled with evidence-based ICT programs using user-centric designs, wearable device, and machine learning approaches could be used to provide personalized interventions for people with diabetes [84,87-93]. Hence, our findings could have practical and research implications for diabetes medication adherence.

### Conclusions

Our framework to evaluate smartphone apps in promoting diabetes medication adherence has considered physiological factors influencing diabetes and app features. Therefore, our findings could have positive implications for the design and development of apps for patients with diabetes. Additionally, the available apps could be evaluated in accordance with our framework, and those apps promoting higher medication adherence could be prescribed for better health outcomes.

## Appendix

Multimedia Appendix 1 Apps Selected for the Review and MASS.

Multimedia Appendix 2 Detailed information on all apps.

## Abbreviations

BG: blood glucose
BGM: blood glucose monitoring
CGM: continuous glucose monitoring
ICT: information and communication technology
MARS: Mobile App Rating Scale
MASS: mean app-specific score
mHealth: mobile health
OS: operating system
T1DM: type 1 diabetes mellitus
T2DM: type 2 diabetes mellitus
TTF: task-technology fit
