# Supplementary material for: Smartphone Apps for Diabetes Medication Adherence: Systematic Review
Source: JMIR Diabetes. 2022 Jun 21;7(2):e33264. doi: 10.2196/33264 (PMC9257622; doi:10.2196/33264)
Supplement: Multimedia Appendix 1 [file diabetes_v7i2e33264_app1.docx]

**Multimedia Appendix 1:**

Apps Selected for the Review and MASS

| App | App-specific rating for medical adherence | | | | | | MASS |
| --- | --- | --- | --- | --- | --- | --- | --- |
|  | Awareness | Knowledge | Attitude | Intention to change | Help-seeking | Behavior change |  |
| Diabetes:M [49] | 4 | 4 | 4 | 4 | 4 | 4 | 4 |
| mySugr - Diabetes Tracker Log [52] | 4 | 4 | 4 | 4 | 4 | 4 | 4 |
| Health2Sync [51] | 4 | 4 | 4 | 4 | 4 | 4 | 4 |
| MyTherapy Pill Reminder [53] | 4 | 4 | 4 | 4 | 4 | 4 | 4 |
| One Drop: Transform Your Life [54] | 4 | 4 | 4 | 4 | 4 | 4 | 4 |
| Glucose Buddy Diabetes Tracker [50] | 4 | 4 | 4 | 4 | 4 | 4 | 4 |
| OneTouch Reveal [55] | 5 | 4 | 5 | 5 | 5 | 5 | 4.8 |
| Sugarmate [56] | 5 | 5 | 5 | 5 | 4 | 5 | 4.8 |
