# Supplementary material for: Smartphone Apps for Diabetes Medication Adherence: Systematic Review
Source: JMIR Diabetes. 2022 Jun 21;7(2):e33264. doi: 10.2196/33264 (PMC9257622; doi:10.2196/33264)
Supplement: Multimedia Appendix 2 [file diabetes_v7i2e33264_app2.docx]

**Multimedia Appendix 2**

| App |  | **Diabetes:M [49]** | **mySugr - Diabetes Tracker Log [52]** | **Health2Sync [51]** | **MyTherapy Pill Reminder [53]** | **One Drop: Transform Your Life [54]** | **Glucose Buddy Diabetes Tracker [50]** | **OneTouch Reveal [55]** | **Sugarmate [56]** |
| --- | --- | --- | --- | --- | --- | --- | --- | --- | --- |
| **App general information** | | | | | | | | | |
| Available |  | Google play, App Store | Google play, App Store | Google play, App Store | Google play, App Store | Google play, App Store | Google play, App Store | Google play, App Store | App Store, Web application, Amazon Alexa. |
| Category |  | Medical | Medical | Medical | Medical | Health & Fitness | Medical | Medical | Medical |
| Seller |  | Sirma Medical Systems AD | mySugr GmbH | H2 Inc. | smartpatient GmbH | Informed Data Systems, Inc | Tom Xu | LifeScan, Inc. | Sugarmate, LLC |
| Age Rating |  | 17+ | 4+ | 4+ | 4+ | 4+ | 4+ | 17+ | 4+ |
| Ratings (App Store, out of 5) |  | 4.5 (61 Ratings) | 4.6 (669 Ratings) | 4.7 (27 Ratings) | 4.8 (106 Ratings) | 4.5 (18.6K Ratings) | 4.8 (20.4K Ratings) | 4.8 (74.4K Ratings) | 4.8 (7.7K Ratings) |
| In-app purchases |  | Yes  $4.99 - $71.99 | Yes  $42.99 - $599.99 | Yes  $4.49 monthly | No | Yes  $19.99 | Yes  $3.99 -59.99 | No | No |
| Languages |  | English, Bulgarian, Catalan, Dutch, German, Portuguese, Russian, Spanish | English, Bulgarian, Czech, Danish, Dutch, Estonian, Finnish, French, German, Greek, Italian, Latvian, Lithuanian, Norwegian Bokmål, Polish, Portuguese, Romanian, Russian, Slovak, Slovenian, Spanish, Swedish, Turkish | English, Japanese, Simplified & Traditional Chinese | English, Arabic, Bulgarian, Catalan, Czech, Danish, Dutch, Finnish, French, German, Greek, Hebrew, Hindi, Hungarian, Italian, Japanese, Korean, Norwegian Bokmål, Norwegian Nynorsk, Persian, Polish, Portuguese, Russian, Simplified Chinese, Spanish, Swedish, Traditional Chinese, Turkish, Ukrainian | English, Arabic, French, German, Italian, Japanese, Portuguese, Russian, Simplified Chinese, Spanish, Traditional Chinese | English, Arabic, Catalan, Czech, Danish, Dutch, Finnish, French, German, Greek, Hebrew, Hungarian, Indonesian, Italian, Japanese, Korean, Malay, Norwegian Bokmål, Polish, Portuguese, Romanian, Russian, Simplified Chinese, Slovak, Spanish, Swedish, Thai, Traditional Chinese, Turkish, Ukrainian, Vietnamese | English, Croatian, Czech, Dutch, French, German, Hungarian, Italian, Japanese, Polish, Portuguese, Slovenian, Spanish, Vietnamese | English, Dutch, French, Italian, Portuguese |
| Data | Identified | Health & Fitness, Contact Info | Health & Fitness, Contact Info, Identifiers, Diagnostics, Location, User Content, Usage Data | Identifiers, Usage Data, Diagnostics | NA | Location, Contact Info, Identifiers, Usage Data, Sensitive Info | Location, Contact Info, Identifiers, Usage Data | Health & Fitness, Contact Info | NS |
|  | Deidentified | Location, Identifiers, Usage Data, Diagnostics | NA | NA | Health & Fitness, Contact Info, Identifiers, Diagnostics, Location, User Content, Usage Data | Health & Fitness, Location, Contact Info, User Content, Identifiers, Usage Data, Sensitive Info, Diagnostics, Other Data | Health & Fitness, Purchases, Location, Contact Info, User Content, Identifiers, Usage Data, Sensitive Info, Diagnostics, Other Data | Identifiers, Usage Data, Diagnostics |  |
| Developer website |  | https://www.diabetes-m.com/ | https://www.mysugr.com/en/ | https://www.health2sync.com/ | https://www.mytherapyapp.com/ | https://onedrop.today/ | https://www.glucosebuddy.com/ | https://www.onetouch.com/ | https://sugarmate.io/ |
| Compatibility (iOS) |  | 9.0 + | 13.2 + | 11.0 + | 13.0 + | 13.0 + | 12.0 + | 13.0 + | 10.3 + |
| **App objectives & free App features** | | | | | | | | | |
| DM Patients |  | T1DM, T2DM, Gestational DM | T1DM, T2DM, Gestational DM | T2DM | Generic | T2DM | Generic | T1DM, T2DM, Gestational DM | Generic |
| Objective |  | Manage DM | Manage DM | Manage DM | Medicine Tracker & Alarm | DM & heart health management | Manage DM | Manage DM | CGM companion |
| BG source |  | Manually log, import from glucometer | Manually log | Manually log, import from glucometer using Health2Sync Smart Cable | Manually log | Manually log, import from glucometer | Manually log | OneTouch Verio Reflect meter & Flex meter | Dexcom G4/G5/G6 |
| Health data tracking |  | glucose, carbs, proteins, fats, calories, insulin | Steps, activity, blood pressure, CGM data, weight, diet, meds, carb intake | Blood sugar, blood pressure, weight | weight, blood pressure, blood sugar levels | blood sugar, medications, meals, activity, weight, blood pressure etc. | blood sugar, medication, A1C, weight, blood pressure, carb | blood sugar, step tracking, carb tracking, activity tracking | blood sugar, step tracking, carb tracking, activity tracking |
| Device integration |  | Dexcom, One Touch, Accu-Chek, Agamatrix, iHealth, Dario, Garmin, Fitbit, Nike+, UP by Jawbone, Misfit, Pebble, Human, Strava, My Fitness Pal, Lose it!, Lark, Weight Watchers, Withings | Accu-Chek Mobile; Accu-Chek Guide; Accu-Chek Instant & Instant Forward | Sync with Health App | NA | Fitbit, Dexcom, One Touch, Accu-Chek, Agamatrix, iHealth, Dario, Garmin, Nike+, Strava, Qardio, My Fitness Pal, Lose it!, Lark, Weight Watchers, Withings, and more | Dexcom | OneTouch Verio Reflect meter & Flex meter | Dexcom G4/G5/G6 |
| **Diabetes medication adherence** | | | | | | | | | |
| Reports | Factors | Blood sugar entries  Medications  Physical activity  Carbohydrates,  Glucose infusion | diet, meds, carb intake, BG levels | trend of latest BG, blood pressure and weight | blood sugar levels, resting heart rate, and more | blood sugar, medications, meals, activity, weight, blood pressure | blood sugar, insulin, weight, blood pressure, A1C | blood sugar with food, insulin, and activity | GMI, bedtime/wake, total insulin, total carbs |
|  | High / low BG | Notification |  | SMS |  |  |  | Notification | SMS: family & friends with GPS coordinates  Call: during sleep |
|  | Overview | day, week, month, and year | Daily, weekly, and monthly | When app launched |  | Daily, weekly, and monthly | Hourly | 14-, 30- and 90-day | Daily |
|  | Share | Medical experts |  | Download | Download |  | Download | care team | Doctor |
| Reminders |  | Glucose checks, meals |  | according to the uploaded information | medications, measurements, or exercise, | glucose checks, blood pressure checks, weigh-ins, medications, exercise, meals |  | Personalized:  patterns, meds, food, exercise |  |
| Assistance |  | Allows doctors, clinicians, nutritionist, to have patient’s info. |  | Tips according to the uploaded information | Personalized treatment tips |  | 12-Week Diabetes Education Plan featuring 5-minute lessons, Meal IQ | Personalized guidance, insight, & encouragement |  |
| Motivation |  |  | Exciting challenges to achieve personal therapy goals | Invite loved ones | Connect with friends and family for encouragement | Supportive community, Set goals & visualise progress |  | Set goals & visualise progress | Real-time stat, interactive graph |
